# Vaccine-Induced Anti-HBs Level in 5-6 Year-Old Malnourished Children

**DOI:** 10.5812/hepatmon.7048

**Published:** 2013-02-03

**Authors:** Mehran Karimi, Ali Raee, Behnam Baghianimoghadam, Mohammad Hossein Fallahzadeh

**Affiliations:** 1 Children Growth Disorders Research Center, Shahid Sadoughi University of Medical Science, Yazd, IR Iran; 2 Department of Pediatrics, Shahid Sadoughi University of Medical Science, Yazd, IR Iran; 3 Research and Clinical Center for Infertility, Shahid Sadoughi University of Medical Science, Yazd, IR Iran; 4 Department of Biostatistics, Shahid Sadoughi University of Medical Sciences, Yazd, IR Iran

**Keywords:** Hepatitis B, Vaccination, Malnutrition, Children

## Abstract

**Background:**

Malnutrition is the most common cause of immune deficiency. It results in reduced secretion of T-cells and B-cell-stimulating factors leading to declining of special immunoglobulins. On the other hand, hepatitis B, as a major world health problem, can be prevented effectively by vaccination. Three doses of hepatitis B virus (HBV) vaccine induce protective levels of anti-hepatitis B surface (anti-HBs) in 95% of healthy children. This level decreases gradually over time.

**Objectives:**

The goal of this study was to assess anti-HBs in malnourished children, who confronted to some degrees of immune deficiency.

**Patients and Methods:**

This is a cross-sectional study conducted during May to August 2010 in therapeutic clinics of Yazd, Iran. Samples were selected simply and consecutively among 5-6 year-old children with a history of three doses of HBV vaccine in infancy. On the basis of World Health Organization’s definition on malnutrition, which considers anthropometric measurements, malnourished children entered the study. Totally 83 cases (37 boys and 46 girls) were gathered and classified into three groups of mild, moderate, and severe malnutrition. One milliliter of venous blood was taken and anti-HBs were tested by enzyme linked immunosorbant assay (ELISA).

**Results:**

Overall, seroprotection rate and geometric mean titer (GMT) of anti-HBs were 60.2% and 15.47 ± 10.92 mIU/mL, respectively. Seroprotection rate was 71.4%, 55.2%, and 72.7% in mild, moderate, and severe malnourished children, respectively. GMT was 30.78 mIU/mL, 12.15 mIU/mL, and 22.95 mIU/mL in these groups, respectively. None of these two indices were significant in these groups (P = 0.471, P = 0.364). Seroprotection rate and GMT were 54.1% and 13.26 ± 11.59 mIU/mL in boys, and 65.2% and 17.5 ± 10.59 mIU/mL in girls, respectively, showing no significant relationship with gender (P = 0.302, P = 0.602). Lowest seroprotection rate was in stunted cases (47.1%) and highest in wasted children (77.8%). This difference also was not significant (P = 0.43).

**Conclusions:**

The seroprotection rate and GMT of anti-HBs observed in this study do not show a high level of immunity. These two indices were not related to severity of malnutrition. We conclude that severity of malnutrition does not affect vaccine-induced antibody level and seroprotection rate; however small sample size in each group of study hinders decisive conclusion. Moreover, GMT and seroprotection rate showed no relationship with type of abnormal anthropometric index, including weight for height, weight for age, and height for age.

## 1. Background

Viral hepatitis is a major worldwide health problem. HBV has a worldwide spread and is highly prevalent at Asia, Africa, Southern Europe, and Latin America (1). It is estimated that 400 million people suffer from chronic hepatitis (2). Several factors like type of vaccine, site, type and dose of injection, compliance of vaccine cold chain, race, genetic, immunity condition, chronic disease, obesity, age, alcohol use, drug abuse, smoking, and stress can influence on immunologic response to HBV vaccine (3-8). HBV vaccine stimulates production of anti-HBs meaning seroconversion and immunologic memory against HBsAg. This memory causes constant protection of antibody against clinical infection (seroprotection). Persistence of this memory can be evaluated by response to booster dose and spot ELISA (that checks capability of lymphocyte B to produce anti-HBs). About 95% of people (even after 5-12 years after first dose of vaccine) have rapid and high elevations of antibody in response to booster doses (9). Evaluation of anti-HBs is the simplest and the most available test which can anticipate possible decrease in protection after vaccination, and can detect need for booster doses. Vaccination has decreased acute and chronic infection and related complications in children (10, 11). In United States, from 1982 (in which the first generation of HBV vaccine was introduced) total incidence of infection has decreased more than a half (2) and incidence of hepatocellular carcinoma in children has decreased about 75% (10). Nutritional status is major influencing factor in immunologic response; and is major factor of immunodeficiency (10). Reason of malnutrition is different in various regions of Iran including improper complementary food preparation, low parents’ nutritional knowledge, tending to formula usage and its bad preparation, childhood disorders especially digestive and respiratory diseases, and presence of illness in parents such as psychological problems and disabilities. (12, 13) Protein energy malnutrition (PEM) causes cellular and humoral immunity and phagocyte function disorders; complement level (except C4), secretary IgA, and cytokine production will decrease (14-19). Deficiency of zinc, selenium, Fe, copper, vitamins A, B, C, E, B6, and Folic acid have important role for immune response in malnutrition (15, 18, 19). Lymph nodes atrophy is a prominent sign in PEM which lowers the size and weight of thymus (nutritional thymectomy) resulting in sensitivity to pathogens, activation of opportunistic infections, and reactivation of viral infections (14, 17, 18, 20). Total lymphocyte count will be lower during PEM, from which the amount of T-lymphocytes (CD3+, CD4+ and CD8+) will decrease, of B-lymphocytes will remain intact, and of null cells will increase (14, 16, 19, 21). Amount of matured and differentiated T-lymphocytes will decrease, and then due to decreased amount of antibody-secreting cells, T-lymphocyte dependent immunoglobulins will decrease, too (17, 18). Antibody related immune response during PEM usually remains intact (16, 22), especially when antigen is supplied in the form of adjuvant or as a substance whom do not need T-cells. Results of several studies indicate normal or high levels of IgM and IgG (16) and high levels of IgE and IgD (22) but results about IgA were inconsistent (16, 17, 22), although it has been shown that level of secretory IgA after injection of antiviral vaccines is low and, to compensate, IgM level will increase in body secretions. Three- dose injection of HBV vaccine provides a safe level of protection in 95% of infants, children, and healthy teenagers. This primary response to vaccine will decrease with age as after 40 years old, it falls to 90% (6). Laboratory and epidemiologic studies indicate that, according to enzyme immunoassay (EIA) and radioimmunoassay (RIA) techniques, the safe serum level of anti HBsAg after vaccination is 10 mIU/ml (1, 8).

## 2. Objectives

In this study, we assessed 5-year immune insolubility of HBV vaccine in malnourished children who had taken complete three doses of vaccine.

## 3. Patients and Methods

This was a cross-sectional study implemented from May to August 2010 in Yazd, Iran. Eighty-three children (37 boys and 46 girls) were chosen by simple selection among 5-6 years old children who were carried out to health and clinical centers in Yazd. Sample size was calculated considering the following parameters: P < 0.05 as significance, test power of 80%, d = 1.5 and based on previous studies S = 2. All included participants had taken 3 doses of recombinant HBV vaccine (batch No: 91B0031, made by Pasteur institute of Iran) at infancy (0, 2, 6 months of age), and their weight for height, weight for age, or height for age were under -1 SD. All children were visited by general practitioner and they did not have any co-infection or co-morbidity.1mL of venous blood sample was collected from the children and serum levels of anti-HBs antibody were detected using ELISA kits (Kit Diasorin BioMedica, Saluugia, Italy). Heights were measured with Seca height gauge at standing position, while head was in Frankfurt position. Weights were measured with Seca scale (by 100 grams precision). Based on definitions, if weights for height, weight for age, or height for age scales are ≤ -1SD, child is at mild, ≤ -2 SD at moderate, and ≤ -3 SD at severe malnutrition (23). Exclusion criteria of this study were: hemodialysis patients, known primary immunodeficiency, patients under chemotherapy, hemophilia patients, and patients who were not breast fed during first 6 months of age.

### 3.1. Statistical Analysis

All gathered data were transferred to SPSS software 15. Mean, median, and Geometric Mean Titer (GMT) were calculated. GMT is more valuable than statistical mean and median for declaration of the mean of data, it has been used for measurement of antibody values and response to vaccines, because it can omit bias effects of very high and very low titers. Because of low cases we have had in our study groups (based on severity of malnutrition), to compare seroprotection, we used G-StatXact software and Fisher’s exact test. GMT was compared between the groups by ANOVA test. Chi-square and T-test were used to compare seroprotection and GMT according to sex and disturbed scales. We had problems for gathering cases because of limited patients in our specific age range.

## 4. Results

Eighty-three children with mean age of 74 ± 8 months were included in the study. Fourteen children (16.9%) had mild, 58 children (69.9%) moderate and 11 children (13.3%) severe malnutrition. About 60.2% of all children (50 out of 83) had seroprotection, and total GMT was 15.47 ± 10.92. Total statistical mean and median were 290.49 ± 130.97 (Max = 1281 and Min = 0.1) and 21, respectively. Seroprotection rate in children with mild, moderate, and severe malnutrition were 71.4%, 55.2%, and 72.7%, respectively. There was no significant difference between the groups (P = 0.471). Amount of GMT was 30.78 mIU/mL, 12.15 mIU/mL, and 22.95 mIU/mL for mild, moderate, and severe malnutrition, respectively. There was no significant difference between the groups according to GMT, too (P = 0.364) (Table 1). Seroprotection and GMT were higher in females than those were in males but differences were not significant (P = 0.302 and P = 0.43, respectively) (Table 2). The lowest rate for seroprotection was for short stature children (47.1%) and the highest for low weights (77.8%) (P = 0.43).

**Table 1:** GMT, Statistical Mean and Median of Anti-HBs and Seroprotection Rate According to Malnutrition Severity

Malnutrition Severity	Mild	Moderate	severe	total	P value
**Anti-HBs level**					
GMT± SD, mIU/mL	30.78 ± 7.18	12.15 ± 11.17	22.95 ± 15.27	15.47 ± 10.93	0.364 a
Mean ± SD	141.64 ± 252.36	118.37 ± 284.79	183.82 ± 290.49	140.97 ± 290.49	
Median	26.9	16	26	21	
**Sero protection rate, %**	10 of 14 (71.4)	32 of 58 (55.2)	8 of 11 (72.7)	50 of 83 (60.2)	0.471 b

Abbreviations: anti-HBs, anti-hepatitis B surface; GMT, geometric mean titer

^a^ANOVA

^b^Fisher`s exact test

**Table 2:** GMT and Statistical Mean of Anti-HBs Level and Seroprotection Rate According to Sex

Sex	Male	Female	P value
**GMT ± SD, mIU/mL**	13.26 ± 11.59	17.50 ± 10.59	0.602 a
**Mean ± SD, mIU/mL**	12.76 ± 297.53	137.68 ± 287.84	
**Seroprotection rate, No. (%)**	20 of 37 (54.1)	30 of 46 (56.2)	0.302 b

Abbreviations: GMT, geometric mean titer

^a^T-test

^b^Chi-square

## 5. Discussion

Our study indicates that in 5-6 years old malnourished children, mean level of anti-HBs was 15.47 mIU/mL, and the rate of seroprotection was 60.2%. Rate of seroprotection in our study was lower than that in Jafarzadeh et al study conducted in Rafsanjan, Iran. In that study, 81.5% (GMT = 206 mIU/mL) of healthy children had anti-HBs seroprotective levels which decreased to 47.9% after 10 years (GMT = 9.6 mIU/mL) (24). Seroprotection rate in different country studies were different; in New Zealand, 85% (25); Ethiopia, 89% (22); Spain, 85% (26); Taiwan, 86% (9); China, 54.9% (27); Egypt, 81% (11); Venezuela, 71% (8); United States, 41% (28); and another study in New Zealand, 56% (29). In Turkey, Karaglu et al studied on 210 children of 1-3 years old who were vaccinated against HBV. They concluded that 96.7% of children were immune and there was no significant correlation between anthropometric scales and level of immunization. accept, sex, and also site, and time of injection were not effective on immune response (30). Rey and colleagues conducted a cross-sectional study in Senegal and Cameroon countries to assess HBV immune protection rates among children. They found that nutritional status was significantly correlated with the response to HBV vaccination (P < 0.001); 85% of children with normal nutrition status were protected (anti-HBs ≥ 10 IU/L) versus 60% in moderate to severe malnutrition. The percentages of protected children in the two countries were lower among children with moderate or severe malnutrition (12% vs 20% in Cameroon, 62% vs 71% in Senegal) (31). Muhammad et al studied the immune response of hepatitis B immunization on infants with 0, 2, 9 and 3, 4, 9 months of age schedules. They found mild malnutrition produced the highest percentage of protective immune response while the non-protective immune response occurred in two infants of well-nourished group and one of mild and moderate malnutrition. They concluded that nutritional status had no influence on anti-HBs level. (32). Generally, antibody titer will be reduced rapidly at the first year of vaccination and slowly, thereafter (8). But Lozano in his study on 5-7 years old children in Madrid had different results and concluded that antibody titer would not decrease with age (33). In two studies, annual titer reduction in children under 7 years old was 10.2% and in 7-16 years old was 20%; GMT had inverse linear relationship with logarithm of time (the slope of the line = -1.6) (10, 34). Now, current studies show that in healthy people, vaccine-induced immune memory will be preserved well. Even if anti-HBs titer becomes zero during the time, long time protection against clinical disease and chronic infection will remain. Any chronic infection was not seen among adults who responded to vaccination; almost all unexpected infections were documented in infants. In immune competent people, even with anti-HBs less than 10 mIU/mL, there is no need to booster doses (8). Jamal et al in his study on 44 infants in Egypt showed that in healthy infants, two months after the last dose of HBV vaccine, rate of protection was 100%, while in protein energy malnourished ones, it was 87%, although difference was not significant (17). In another study in Egypt on 200 children, there was no difference in growth and nutrition (evaluated by height, weight, mid-arm circumference, and serum albumin) of children with either anti-HBs titer ≥ 10 or ≤ 10 mIU/mL (11). These seemingly contradictory findings in survival of vaccine can be due to racial differences or non-apparent exposure to HBV in endemic areas (11, 34). These differences stress on the necessity of more studies with the higher sample size and consideration of possible factors such as age, sex, race, site of injection, nutritional status, vaccine brand, and calibration of kits. In most of previous studies, participants were not allocated based on their height or weight, and therefore, undiagnosed malnourished children were not excluded from studies. It is necessary to include a control group (children without malnutrition) in future studies. In our study, there was no correlation of severity of malnutrition and rate of seroprotection with anti-HBs (Geometric Mean Titer (GMT)); also, severity of malnutrition had no effect on anti-HBs levels and seroprotection rate. We may postulate that malnutrition in children cannot prevent a competent immune response to HBV vaccine. In our study, there was no significant correlation between GMT of anti-HBs, seroprotection, sex and type of disturbed scales (weight for height or BMI, weight for age, and height for age) in malnourished children. Given the small sample size in each of three groups, conclusion based on the severity of malnutrition is not possible, firmly. Aria et al in his study has shown negative effect of malnutrition, immune deficiency, and drug addiction on HBV vaccine immune response (3). Wang in Taiwan has concluded that the response to HBV vaccine in low socio-economic areas was lower (35). Another study showed that low BMI, smoking, and some specific races had adverse effect on HBV immune response, but low weight and short stature did not (36). In a study on rats in Japan, results showed that most of rats with PEM did not respond to HBV immunization, while all rats in control group responded. In a histopathology study, PEM caused a decrease in dendritic cells and their stimulatory functions on T cells (producing IL12P70 and IFNγ) (37). Obesity is a type of malnutrition (38); some studies showed that obesity was a predicting factor for weak response to HBV vaccine (39, 40). Frequent studies surveyed the effect of malnutrition in response to HBV vaccine in hemodialysis patients. In some of these studies, impact of malnutrition on seroconversion has been shown (39, 40) but some others found no effect (13, 41-44). Based on our study, severity of malnutrition has no effect on seroprotection rate and mean level of anti-HBs after HBV vaccination in 5-6 years old children, and there is no need to additional booster doses or periodic laboratory rechecks. Although these results are certain for malnutrition as a whole, our small sample size makes us impossible to conclude definitively about severity of malnutrition. More researches with larger sample size are required helping us for better judgment. Also studies on people who were infected with HBV despite HBV vaccination and searching about their nutritional status can help us to answer this question with more confidence.
